# Chain mediating effect of social support and psychological resilience between quality of discharge teaching and readiness for hospital discharge in patients with diabetic retinopathy surgery

**DOI:** 10.3389/fpubh.2026.1820072

**Published:** 2026-06-10

**Authors:** Yuanyuan Wang, Qianqian Zhang, Haosen Pan, Hong Liu, Ankai Chen, Maoyun Miao

**Affiliations:** 1 The Ophthalmology Center, Affiliated Hospital of Shandong Second Medical University, Weifang, China; 2 School of Nursing, Shandong Second Medical University, Weifang, China

**Keywords:** diabetic retinopathy, mediating effect, psychological resilience, quality of discharge teaching, readiness for hospital discharge, social support

## Abstract

**Background:**

With the increasing incidence of diabetic retinopathy (DR) and shortened hospital stays, postoperative home rehabilitation for patients faces significant challenges, making improved readiness for hospital discharge a key focus of ophthalmic nursing. This study aims to explore the specific pathways through which the quality of discharge teaching is associated with readiness for hospital discharge in DR surgery patients, and to analyze the chain-mediating role of social support and psychological resilience, providing a theoretical basis for developing targeted clinical nursing interventions.

**Methods:**

Using a convenience sampling method, 489 patients undergoing surgery for DR at a tertiary Grade A hospital in Weifang, Shandong Province, were recruited between July 2025 and January 2026. Data were collected via a cross-sectional survey utilizing the General Information Questionnaire, the Readiness for Hospital Discharge Scale (RHDS), the Quality of Discharge Teaching Scale (QDTS), the Social Support Rating Scale (SSRS), and the Connor-Davidson Resilience Scale (CD-RISC). Structural equation modeling (SEM) was employed to test the hypothesized chain-mediating pathway.

**Results:**

The mean scores for quality of discharge teaching, readiness for hospital discharge, social support, and resilience were 114.24 ± 26.92, 74.47 ± 18.97, 40.00 ± 8.13, and 59.97 ± 19.35, respectively. Quality of discharge teaching was positively correlated with social support, psychological resilience, and readiness for hospital discharge (*p* < 0.01). Mediation analysis indicated that social support and psychological resilience significantly mediated the relationship between quality of discharge teaching and readiness for hospital discharge through a chain-mediating pathway. This mediating pathway accounted for 36.38% of the total association between quality of discharge teaching and readiness for hospital discharge.

**Conclusion:**

The quality of discharge teaching is directly associated with readiness for hospital discharge and is indirectly related to it through the mediating roles of social support and psychological resilience. Nursing interventions should focus on enhancing social support systems and fostering psychological resilience to optimize readiness for hospital discharge in patients following DR surgery.

## Introduction

Diabetes mellitus has emerged as a global public health crisis. According to the 2021 International Diabetes Federation (IDF) report, the global prevalence of diabetes among adults aged 20–79 years reached 537 million, with projections suggesting an increase to 783 million by 2045, representing approximately 12.2% of the adult population (1). Diabetic retinopathy (DR) is among the most prevalent and debilitating microvascular complications of diabetes, characterized by microaneurysms, hemorrhage, exudation, and neovascularization (2). It remains the primary cause of vision loss and blindness among the working-age population globally (3). Statistical evidence indicates that approximately 25% of the global diabetic population is affected by DR, with 6% progressing to vision-threatening stages (4). Beyond impairing visual function and quality of life, DR significantly complicates postoperative disease management and home-based care (5). Patients with visual impairment often struggle to read medication instructions, monitor blood glucose accurately, recognize early warning signs of complications, and adhere to complex rehabilitation regimens, increasing their dependence on caregivers and the risk of postoperative complications (6). For patients presenting with severe vitreous hemorrhage or tractional retinal detachment, Pars Plana Vitrectomy (PPV) serves as the definitive surgical intervention (7). While PPV is effective in preserving vision, patients often face a protracted recovery period characterized by slow visual restoration, impaired self-care capacity, and a complex rehabilitation regimen (8). Under the paradigms of Enhanced Recovery After Surgery (ERAS) and the increasing prevalence of day-surgery models, hospital stays have been markedly shortened. Consequently, patients are frequently discharged before achieving full physiological recovery, yet their visual impairment may limit their ability to understand and implement discharge instructions independently, making their rehabilitation heavily dependent on post-discharge self-management and familial support (9). These challenges underscore the critical importance of optimizing readiness for hospital discharge, not only to ensure safe transition to home but also to support effective self-care in patients facing visual and cognitive barriers. Current nursing interventions, however, remain largely focused on the perioperative phase, often lacking the systematic, personalized frameworks necessary for continuous quality of discharge teaching. This gap is associated with an elevated risk of postoperative complications and unplanned readmissions (10). Thus, optimizing readiness for hospital discharge within a limited clinical window has become an urgent priority in ophthalmic nursing.

Readiness for hospital discharge is defined as a clinician’s comprehensive assessment of a patient’s physical, psychological, and social capacity to transition from the hospital to a home-based rehabilitation environment (11). It serves as a pivotal metric for evaluating the continuity of care between inpatient services and community recovery (12). Research indicates that higher readiness for hospital discharge correlates with improved self-management, superior rehabilitation outcomes, and lower readmission rates (13, 14). While the quality of discharge teaching is a key factor associated with readiness for hospital discharge—providing the health education and support necessary to reduce patient uncertainty (15–17)—clinical observation suggests that the association is not limited to a direct path. Some patients report low readiness for hospital discharge despite receiving standard teaching, indicating that individual psychological and social resources likely play a potential mediating role in this association.

The Stress and Coping Theory, proposed by Lazarus and Folkman, posits that an individual’s adaptive outcome to health-related stress is not solely determined by the stressful event itself (18). Rather, it manifests through the cognitive appraisal of the event and the subsequent mobilization and utilization of internal psychological and external environmental resources. Furthermore, Kumpfer’s Resilience Framework elucidates that positive adaptation is not a static individual trait, but rather a dynamic process associated with the interaction between individual characteristics and environmental factors within a risk context (19). Crucially, protective factors in the external environment are considered essential prerequisites for fostering and activating psychological resilience. Within the context of this study, coping with the stress of postoperative DR rehabilitation necessitates the synergistic mobilization of both external and internal resources. Social support, derived from networks of spouses, relatives, and peers, provides the emotional and instrumental resources essential for coping with post-surgical challenges (20, 21). Concurrently, psychological resilience reflects a patient’s internal capacity to achieve positive adaptation in the face of medical adversity (22, 23). In chronic disease management, robust social support fosters a sense of security and control, which in turn nurtures psychological resilience (24). Therefore, this study hypothesizes that high-quality of discharge teaching is not only positively associated with readiness for hospital discharge, but also positively associated with perceived social support by empowering the patient’s social support system. This robust external support is subsequently associated with greater psychological resilience, ultimately forming a potential chain-mediation model to readiness for hospital discharge.

Despite its importance, the complex structural interrelationships linking these four variables have not been fully elucidated in the context of DR surgery (25, 26). This study aims to construct and validate a chain-mediation model to elucidate these pathways, thereby providing a theoretical foundation for optimizing nursing interventions and improving the transition from hospital to home for patients undergoing surgery for DR.

## Method

### Study design

This study employed a cross-sectional design. Participants were recruited via convenience sampling from the Department of Ophthalmology at a tertiary Grade A hospital in Weifang, Shandong Province, between July 2025 and January 2026.

### Inclusion and exclusion criteria

Patients were eligible for inclusion if they met the following criteria: (1) diagnosed with DR according to the Chinese Clinical Guidelines for the Diagnosis and Treatment of DR (27); (2) underwent PPV; (3) aged 18 years and above; and (4) demonstrated no cognitive impairment, with the capacity to understand and communicate effectively and complete the survey independently. Exclusion criteria included: (1) presence of major organ dysfunction (e.g., heart, liver, kidney, or lung failure); (2) clinical diagnosis of cognitive impairment or acute confusion; and (3) concurrent malignant tumors or other life-threatening comorbidities.

### Sample size estimation

According to the Kendall’s sample size estimation method, the sample size should be 5 to 10 times the number of items. Given that this study involved 29 independent variables/items, the minimum required sample size ranged from 145 to 290. To account for a potential 20% attrition rate or invalid responses, the target sample size was adjusted to 174–348. The final sample of 489 patients exceeded this requirement, ensuring sufficient statistical power for structural equation modeling (SEM).

### Ethical considerations

This study was conducted in accordance with the Declaration of Helsinki and received formal approval from the Medical Ethics Committee of the study hospital (Approval No. SDSMU-2025-KY-218). All participants provided informed consent prior to data collection, and they were assured of the confidentiality and anonymity of their responses.

### Instruments

#### General information

General information questionnaire was designed by researchers, covering gender, age, education level, marital status, occupation, family monthly income per capita, payment method of medical expenses, smoking history, drinking history, family history of diabetes, current residence, main caregivers, duration of diabetes, number of hospitalization due to DR, treatment methods of diabetes and frequency of blood glucose monitoring.

#### Readiness for hospital discharge

The Readiness for Hospital Discharge Scale (RHDS) was evaluated by the Chinese version of the RHDS scale translated by Lin et al. (28). The scale included 3 dimensions of personal status, adaptive capacity and expected support, with a total of 13 items. The first item is a judgment question, which is not included in the total score. The remaining items were scored from 0 to 10 points, and the total score ranged from 0 to 120 points, with higher scores indicating higher readiness for hospital discharge. The total score < 72 was considered as low level, 72–96 as medium level, and >96 as high level. The overall Cronbach’s *α* coefficient of the scale was 0.89, and the content validity index was 0.88.

#### Quality of discharge teaching

Quality of Discharge Teaching Scale (QDTS) was evaluated by Wang et al. (29) Chinese version of QDTS. It covered four dimensions of expected need content, actual obtained content, guidance skills and effects, and poor content, with a total of 24 items. Each item was scored on a scale of 0 to 10, and the total score was the sum of the scores of “actual content” and “guidance skills and effects”, ranging from 0 to 180. A total score of <108 was considered as low quality, 108 to 144 as medium quality, and >145 as high quality. The overall Cronbach’s *α* coefficient of the scale was 0.924.

#### Social support

Social Support rating Scale (SSRS) adopted the SSRS developed by Xiao (30), including three dimensions of subjective support, objective support and social support utilization, a total of 10 items. The total score ranged from 12 to 66, with higher scores indicating higher levels of social support. The Cronbach’s *α* coefficient of the scale was 0.782.

#### Psychological resilience

Connor-Davidson Resilience Scale (CD-RISC) was used in the Chinese version of CD-RISC by Yu and Zhang et al. (31), which included 3 dimensions of strength, optimism and tenacity, with a total of 25 items. Likert 5-point scale (0–4 points) was used, and the total score was 0–100 points. The higher the score, the better the psychological resilience. The Cronbach’s *α* coefficient of the scale was 0.910.

#### Data collection and quality control methods

A survey team composed of two nursing graduate students who had received unified training was responsible for explaining the purpose and process of the study to patients, obtaining informed consent and conducting the survey. The general information of the patients was obtained by reviewing the medical records and the questionnaire, which was filled out by the patients independently within 4 h before discharge. The investigators used unified instructions to explain the requirements of filling in the questionnaire, answered the questions on the spot and collected the questionnaire immediately. All surveys were conducted in a quiet environment to minimize interference.

### Statistical analysis

Excel was used for double data entry and SPSS 26.0 was used for statistical analysis. Continuous variables were described as the mean ± standard deviation, while categorical variables were described as the number of cases and percentage. Independent sample t test was used for data conforming to normal distribution and homogeneity of variance. Pearson correlation analysis was used to explore the correlation between social support, psychological resilience, quality of discharge teaching and readiness for hospital discharge in patients with DR surgery. AMOS 24.0 was used for confirmatory factor analysis (CFA), structural equation model test and Bootstrap mediation analysis. Bootstrapping with 5,000 iterations was performed; a 95% confidence interval (CI) that did not include 0 indicated a significant indirect association. *p* < 0.05 was considered as statistically significant.

## Results

### General information of respondents

A total of 507 questionnaires were distributed, of which 489 valid responses were returned, yielding an effective response rate of 96.45%. The final cohort comprised 489 patients who underwent surgical intervention for DR. The participants were predominantly male (52.8%) and unmarried (42.7%). Regarding educational attainment, the largest proportions of the sample had completed junior high school (37.4%) or college or above (30.5%), while 21.9% had a primary school or below and 10.2% held a senior high school. Approximately 27.4% of the participants were public servant. In terms of lifestyle and clinical history, most participants were never or former smokers (61.8%) and never or former drinkers (78.3%), with 73.2% reporting no family history of diabetes. Regarding the duration of diabetes, 58.1% of the cohort had been living with the condition for ≤10 years (21.5% for <5 years; 36.6% for 6–10 years), whereas 30.1% reported a disease duration exceeding 15 years. Detailed demographic and clinical characteristics are summarized in Table 1.

**Table 1:** General information of patients with diabetic retinopathy surgery (*n* = 489).

Item	Category	*n* (%)
Gender	Male	258 (52.8)
	Female	231 (47.2)
Age	18–44	228 (46.6)
	45–59	113 (23.1)
	≥60	148 (30.3)
Educational level	Primary school or below	107 (21.9)
	Junior high school	183 (37.4)
	Senior high school/Technical secondary school	50 (10.2)
	College or above	149 (30.5)
Marital status	Unmarried	209 (42.7)
	Married	176 (36)
	Divorced/Widowed	104 (21.3)
Occupation	Farmer	70 (14.3)
	Self-employed	122 (24.9)
	Worker	96 (19.6)
	Public servant	134 (27.4)
	Retired	42 (8.6)
	Unemployed/Other	25 (5.1)
Monthly family income (CNY)	0–1,000	108 (22.1)
	1,001–3,000	184 (37.6)
	3,001–5,000	124 (25.4)
	>5,000	73 (14.9)
Medical expense payment method	Self-payment	59 (12.1)
	Medical insurance	430 (87.9)
Smoking status	Never/Former smoker	302 (61.8)
	Current smoker	187 (38.2)
Alcohol consumption status	Never/Former drinker	383 (78.3)
	Current drinker	106 (21.7)
Family history of diabetes	No	358 (73.2)
	Yes	131 (26.8)
Current residence	Rural area	249 (50.9)
	Urban area	240 (49.1)
Primary caregiver	Self	267 (54.6)
	family	222 (45.4)
Duration of diabetes	<5 years	105 (21.5)
	6–10 years	179 (36.6)
	11–15 years	58 (11.9)
	>15 years	147 (30.1)
Hospitalization times per year	1	81 (16.6)
	2	168 (34.4)
	3	157 (32.1)
	≥4	83 (17)
Antidiabetic treatment	Oral hypoglycemic agents only	252 (51.5)
	Insulin injection only	88 (18)
	Oral hypoglycemic agents + Insulin	149 (30.5)
Blood glucose monitoring frequency	Daily	207 (42.3)
	Weekly	171 (35)
	Monthly or less frequently	111 (22.7)

### Scores of patients’ readiness for hospital discharge, quality of discharge teaching, social support, and psychological resilience

The descriptive statistics for the primary research variables are summarized in Table 2. Readiness for Hospital Discharge and Quality of Discharge Teaching. The total score for readiness for hospital discharge was 74.47 ± 18.97. Regarding the quality of discharge teaching, the total score was 114.24 ± 26.92. The social support rating total score was 40.00 ± 8.13 and the total score for psychological resilience was 59.97 ± 19.35. Furthermore, the absolute values of skewness for all primary variables ranged from 0.076 to 0.174, and the absolute values of kurtosis ranged from 0.21 to 0.781. These values are well within the acceptable limits (absolute skewness < 3 and absolute kurtosis < 10), indicating that the data did not deviate significantly from a normal distribution and met the prerequisites for subsequent parametric statistical analyses.

**Table 2:** Descriptive statistics and reliability analysis of each scale (*n* = 489).

Variables	Score (^−^*X* ± *S*)	Skewness	Kurtosis	Cronbach’s α
Readiness for hospital discharge	74.47 ± 18.97	−0.076	−0.759	0.923
Quality of discharge teaching	114.24 ± 26.92	−0.090	−0.532	0.957
Social support	40.00 ± 8.13	−0.147	−0.21	0.842
Psychological resilience	59.97 ± 19.35	−0.101	−0.781	0.959

### Correlation between quality of discharge teaching, readiness for hospital discharge, social support and resilience

Pearson correlation analysis was performed to evaluate the interrelationships among the quality of discharge teaching, social support, psychological resilience, and readiness for hospital discharge. As summarized in Table 3, all variables exhibited significant positive correlations (*p* < 0.001). Specifically, quality of discharge teaching demonstrated strong positive associations with readiness for hospital discharge (*r* = 0.671), psychological resilience (*r* = 0.500), and social support (*r* = 0.599). Furthermore, social support was significantly correlated with both psychological resilience (*r* = 0.430) and readiness for hospital discharge (*r* = 0.558). Finally, a robust positive correlation was observed between psychological resilience and readiness for hospital discharge (*r* = 0.589). These findings suggest that higher-quality of discharge teaching and psychosocial resources are closely linked to higher patient readiness for hospital discharge.

**Table 3:** Correlation coefficient among quality of discharge teaching, readiness for hospital discharge, social support and psychological resilience of patients (*n* = 489).

Variables	Quality of discharge teaching	Rating of social support	Psychological resilience	Readiness for hospital discharge
Quality of discharge teaching	1			
Social support	0.599**	1		
Psychological resilience	0.500**	0.430**	1	
Readiness for hospital discharge	0.671**	0.558**	0.589**	1

**p < 0.001.

### Assessment of the measurement model and multicollinearity diagnostics

Before constructing the structural equation model, the measurement model was evaluated to assess multicollinearity, model fit, and validity. The multicollinearity diagnostics (Table 4) indicated that all predictor variables had variance inflation factor (VIF) values ranging from 1.383 to 1.757, with tolerance values between 0.569 and 0.723, suggesting that multicollinearity was not a concern. CFA was then conducted to examine the measurement model’ s fit (Table 5), and the results demonstrated a very good fit, with all indices meeting recommended thresholds: *χ*^2^/df = 1.964, RMSEA = 0.044, GFI = 0.969, AGFI = 0.949, IFI = 0.988, TLI = 0.983, and CFI = 0.988. Regarding convergent validity (Table 6), the average variance extracted (AVE) for all latent variables ranged from 0.543 to 0.743, and composite reliability (CR) ranged from 0.780 to 0.897, both exceeding the commonly recommended thresholds of 0.50 and 0.70, respectively. Finally, discriminant validity analysis (Table 7) showed that the square root of the AVE for each construct (0.737–0.862) was higher than its correlations with other variables, confirming that the constructs were distinct.

**Table 4:** Multicollinearity diagnostics.

Dependent variable	Predictor variable	VIF	Tolerance
Psychological resilience	Quality of discharge teaching	1.559	0.642
	Social support	1.559	0.642
Readiness for hospital discharge	Quality of discharge teaching	1.757	0.569
	Social support	1.616	0.619
	Psychological resilience	1.383	0.723

**Table 5:** Model fit results of confirmatory factor analysis.

Fit indices	Criterion	Current value
*χ* ^2^	–	94.267
df	–	48
*χ*^2^/df	<3	1.964
RMSEA	<0.08	0.044
GFI	>0.90	0.969
AGFI	>0.90	0.949
NFI	>0.90	0.975
RFI	>0.90	0.966
IFI	>0.90	0.988
TLI	>0.90	0.983
CFI	>0.90	0.988
PNFI	>0.50	0.709
PCFI	>0.50	0.718

**Table 6:** Convergent validity test results of the measurement model.

Variables	AVE	CR
Quality of discharge teaching	0.740	0.895
Social support	0.543	0.780
Psychological resilience	0.743	0.897
Readiness for hospital discharge	0.693	0.871

**Table 7:** Discriminant validity test results.

Variables	Quality of discharge teaching	Social support	Psychological resilience	Readiness for hospital discharge
Quality of discharge teaching	0.860			
Social support	0.599	0.737		
Psychological resilience	0.500	0.430	0.862	
Readiness for hospital discharge	0.671	0.558	0.589	0.832

### Mediating pathways of patients’ social support and psychological resilience between quality of discharge teaching and readiness for hospital discharge

**Figure 1:**
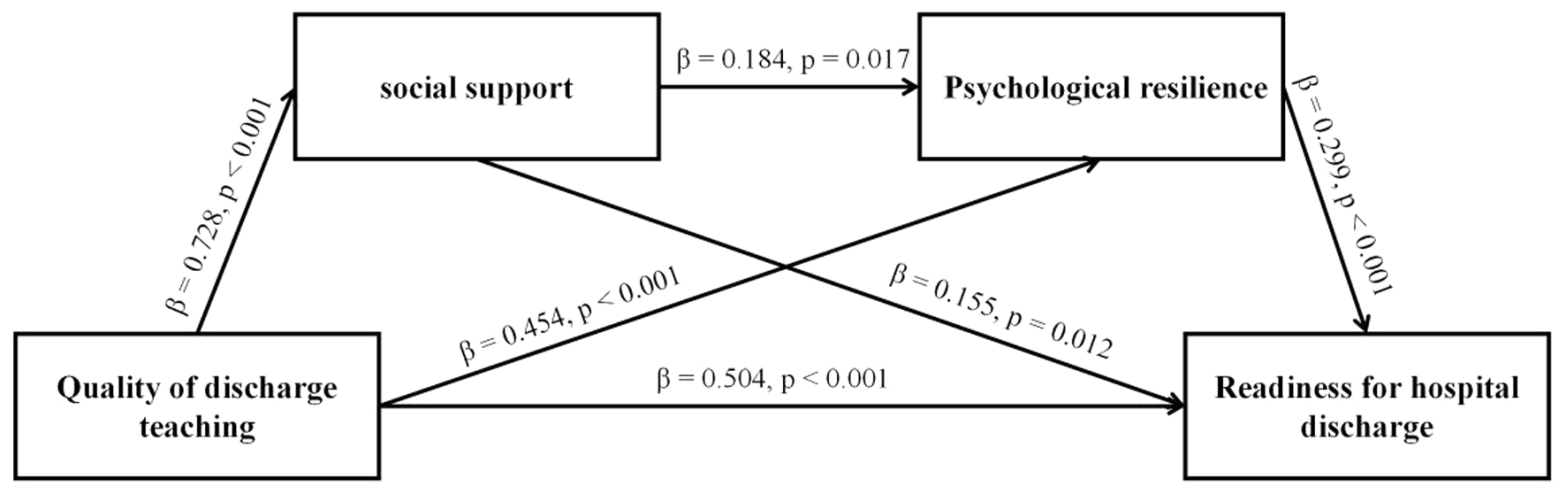
Chain mediating model of social support and resilience on the quality of discharge teaching and readiness for hospital discharge.

The structural equation model was constructed with readiness for hospital discharge as the dependent variable, social support and psychological resilience as mediating variables, and quality of discharge teaching as the independent variable (Figure 1). Path analysis revealed that the quality of discharge teaching was significantly and positively associated with social support (*β* = 0.728, *p* < 0.001), psychological resilience (*β* = 0.454, *p* < 0.001), and readiness for hospital discharge (*β* = 0.504, *p* < 0.001). Social support was positively associated with psychological resilience (*β* = 0.184, *p* = 0.017) and readiness for hospital discharge (*β* = 0.155, *p* = 0.012), while psychological resilience also had a positive association with readiness for hospital discharge (*β* = 0.299, *p* < 0.001). These results indicate that social support and psychological resilience play a chain mediating role between the quality of discharge teaching and readiness for hospital discharge. As shown in Table 8, bootstrap analysis (5,000 iterations) confirmed the significance of these mediating pathways. The total indirect effect was 0.215, accounting for 36.38% of the total effect, whereas the direct effect was 0.376, accounting for 63.62% of the total effect.

**Table 8:** Bootstrap tests for mediating effects and effect values.

Type of effect	Path	Unstandardized effect	Boot standard error	Bootstrap 95% CI		Proportion of Effect
				LLCI	ULCI	
Direct Effect	Quality of discharge teaching → Readiness for hospital discharge	0.376	0.028	0.234	0.345	63.62%
Indirect Effect	Quality of discharge teaching → Social support → Readiness for hospital discharge	0.084	0.035	0.019	0.156	14.20%
	Quality of discharge teaching → Psychological resilience → Readiness for hospital discharge	0.101	0.024	0.060	0.154	17.09%
	Quality of discharge teaching → Social support → Psychological resilience → Readiness for hospital discharge	0.030	0.014	0.006	0.061	5.10%
Total Indirect Effect	Quality of discharge teaching → Readiness for hospital discharge	0.215	0.04	0.139	0.298	36.38%
Total Effect	Quality of discharge teaching → Readiness for hospital discharge	0.591	0.033	0.529	0.661	100.0%

## Discussion

The present study demonstrated that the overall readiness for hospital discharge of patients undergoing DR surgery is at a moderate level, which is consistent with the findings of Qiu et al. (15) regarding patients undergoing day surgery for cataracts. This study reflects that ophthalmic surgery patients have certain common characteristics in readiness for hospital discharge. Patients are generally able to complete basic readiness for hospital discharge. Nevertheless, this level of readiness remains significantly insufficient for coping with the complex, long-term self-management tasks required following DR surgery. The multifaceted nature of postoperative rehabilitation is associated with the inherent complexity of readiness for hospital discharge. As a chronic progressive complication of diabetes mellitus (32), the postoperative rehabilitation of DR involves not only ocular recovery but also more stable and long-term blood glucose control, improved medication compliance, and appropriate lifestyle adjustments (33). Following discharge, patients must simultaneously manage multidimensional tasks: first, ensuring postoperative ocular recovery by avoiding complications (e.g., infection, hemorrhage) and promoting stable visual function. Second, maintaining systemic metabolic control, as blood glucose stability is the cornerstone of ocular healing and long-term surgical efficacy. Third, adhering strictly to medication regimens, which requires coordinating and monitoring both antidiabetic and ophthalmic drugs. Fourth, making continuous lifestyle adjustments, including dietary modifications and appropriate exercise. This multi-dimensional management system requires high levels of extremely high requirements for patients’ disease knowledge, operational skills and behavior persistence. At present, there is a significant cognitive and behavioral gap between patients in this multi-dimensional management system. Studies have shown (34) that many DR Patients have insufficient knowledge on how to identify the association between postoperative ocular symptoms and blood glucose fluctuations and how to coordinate different drug treatment regimens. At the same time, the burden of long-term disease management and concerns about long-term prognosis also weaken patients’ confidence in rehabilitation and self-efficacy (35), which in turn may be negatively associated with their willingness and ability to continue effective self-management after discharge. The moderate readiness for hospital discharge observed in this cohort suggests potential limitations of quality of discharge teaching when applied to patients managing complex, concurrent chronic diseases. Therefore, it is necessary to build a more integrated and continuous readiness for hospital discharge plan, to go beyond the category of general guidance, and optimize the transitional care for patients from hospital to home, and improve the long-term rehabilitation effect.

This study found that the quality of discharge teaching, social support and resilience were all significantly and positively correlated with readiness for hospital discharge. These findings suggest that readiness for hospital discharge is not merely linked to a single disease or surgical factor, but rather demonstrates complex associations with multidimensional factors. Quality of discharge teaching may serve as a primary channel for patients to obtain health information and nursing support, and its level is significantly associated with patients’ understanding and mastery of postoperative care points, early warning signals of complications, and long-term management requirements (36). Previous studies have shown that high-quality discharge teaching can help patients and their families clearly understand the key points of long-term management such as postoperative eye care, blood glucose monitoring, drug use, and complication identification, so as to is associated with improved adaptability after discharge (37). However, the visual limitations of DR patients in the early postoperative period may prevent “quality of discharge teaching” from effectively associating with “readiness for hospital discharge.” Text-heavy written instructions are often insufficient for these patients. Therefore, discharge education should adopt a multimodal approach. Nurses should prioritize oral teaching using simplified language and the “teach-back” method to ensure comprehension. Written materials should be visually optimized, using large print and high-contrast formats. Audio-based digital resources, such as QR codes linking to audio instructions, can provide essential recovery information without relying on vision. Finally, considering the mediating role of social support, education should involve a “nurse–patient–caregiver” model, training primary caregivers to compensate for patients’ sensory limitations. Social support also plays an important role in the readiness for hospital discharge of patients with DR surgery. Patients with DR surgery often face the multiple pressures of vision impairment, frequent medical visits and strict self-management. Practical help and emotional support from family, friends and community can not only provide necessary care assistance, alleviate practical difficulties, but also reduce psychological isolation and anxiety of patients, and enhance their motivation and resilience to cope with long-term disease management, which is linked to higher levels of post-discharge adaptability (38). The positive correlation between resilience and readiness for hospital discharge further suggests that resilience plays a non-negligible role in the postoperative recovery of chronic diseases. Patients with high psychological resilience are more likely to respond to the uncertainties and challenges brought by the disease in a positive way and have a stronger adaptability when facing difficulties in the rehabilitation process (23). The results of this study are consistent with previous research (23, 39) on cataract surgery patients, suggesting that nursing staff should assess the level of psychological resilience of patients upon admission, incorporate the improvement of psychological capital into the systematic intervention plan, and enhance the internal strength of patients through structured training to improve their readiness for hospital discharge and long-term adaptability.

The results of this study showed that the quality of discharge teaching has a direct positive association with the readiness for hospital discharge of patients with DR surgery. At the same time, social support and psychological resilience also play a mediating role between the quality of discharge teaching and readiness for hospital discharge of patients with DR surgery. The quality of discharge teaching is a crucial factor associated with readiness for hospital discharge (40). High-quality discharge teaching is associated with higher patient compliance with rehabilitation training and regular follow-ups, serving as an effective safeguard for self-management. Furthermore, professional coaching techniques and effective communication methods foster a cooperative physician-patient partnership. This partnership expands patients’ learning avenues and stimulates their initiative to master disease knowledge and self-management skills, thereby contributing to higher readiness for hospital discharge (39). These findings are closely aligned with the Stress and Coping Theory proposed by Lazarus and Folkman (18). According to this framework, the quality of discharge teaching facilitates a more accurate cognitive appraisal of the post-surgical recovery process, enabling patients to effectively mobilize both external environmental resources (social support) and internal resources (psychological resilience) to cope with the stress of rehabilitation. Specifically, the strong association between quality of discharge teaching and social support observed in this study suggests that professional nursing interventions effectively bolster the patient’s external support system (41). This, in turn, supports Kumpfer’s Resilience Framework (19), which posits that external protective factors are essential prerequisites for activating a patient’s internal psychological resilience. By providing assistance and emotional companionship, social support can reduce the burden of adaptation of patients after discharge and improve their coping confidence, so as to effectively improve the readiness for hospital discharge (42). This robust external support subsequently fosters psychological resilience, which represents an important internal pathway linking the quality of discharge teaching to readiness for hospital discharge (39, 43). Greater psychological resilience, in turn, may enable patients to adapt more successfully to the transition from hospital to home, engage more actively in self-care, and ultimately reach a higher level of readiness for hospital discharge (42). Based on these theoretical mechanisms and study findings, it is recommended to establish a multidisciplinary comprehensive management model led by ophthalmology, in collaboration with endocrinology, diabetes specialist nurses, and community health services, covering all pathways (44, 45). Clinical nursing staff should evaluate patients’ disease cognition, psychological resilience, and social support upon admission. In the short timeframe of a day-surgery model, nurses can promote psychological resilience through practical strategies. For example, digital support tools, such as mobile health apps or WeChat mini-programs, can deliver short educational videos and self-management tips that patients can access anytime. Additionally, peer support networks, connecting patients with others who have successfully managed DR recovery, can offer quick emotional support and practical guidance. Visual education and individualized guidance should be implemented during the perioperative period to help understand the relationship between surgery, blood glucose control, and eye health. Furthermore, pre-discharge interventions must focus on strengthening medication management, blood glucose monitoring, and the self-care skills necessary to identify complications. Finally, clear instructions regarding follow-up appointments and emergency contact procedures must be provided.

This study had several limitations. First, the use of convenience sampling from a single tertiary hospital restricts the representativeness of the sample, which limits the generalizability of our findings to broader populations or different clinical settings. Specifically, the socioeconomic profile of the Weifang region—characterized by a high proportion of agricultural workers and strong traditional family values—may have shaped participants’ perceptions of social support. In this context, robust familial networks may lead to higher perceived social support compared with more urbanized populations, which could have influenced the mediating role of social support in our model. Therefore, caution is warranted when generalizing these findings to other cultural or economic settings. Second, the cross-sectional design precludes the establishment of definitive causal relationships among the variables. Importantly, while SEM and mediation analysis were utilized to explore theoretical pathways, these statistical techniques applied to cross-sectional data cannot verify the temporal sequence of the variables. Consequently, future research should transition from cross-sectional assessments to longitudinal tracking designs. Specifically, it is essential to investigate whether higher readiness for hospital discharge scores serve as a reliable predictor of objective clinical recovery, such as reducing 30 days unplanned readmission rates and the incidence of postoperative complications.

## Conclusion

The results of this study showed that the quality of discharge teaching demonstrated a direct association with the readiness for hospital discharge of patients undergoing DR surgery. Furthermore, the quality of discharge teaching is indirectly associated with readiness for hospital discharge through the chain-mediating roles of social support and psychological resilience.

## Data Availability

The raw data supporting the conclusions of this article will be made available by the authors, without undue reservation.
